# Electronic health records and stratified psychiatry: bridge to precision treatment?

**DOI:** 10.1038/s41386-023-01724-y

**Published:** 2023-09-04

**Authors:** Adrienne Grzenda, Alik S. Widge

**Affiliations:** 1 Department of Psychiatry & Biobehavioral Sciences, David Geffen School of Medicine, University of California—Los Angeles, Los Angeles, CA USA; 2 Olive View-UCLA Medical Center, Sylmar, CA USA; 3 Department of Psychiatry & Behavioral Sciences, University of Minnesota, Minneapolis, MN USA

**Keywords:** Predictive markers, Translational research

## Abstract

The use of a stratified psychiatry approach that combines electronic health records (EHR) data with machine learning (ML) is one potentially fruitful path toward rapidly improving precision treatment in clinical practice. This strategy, however, requires confronting pervasive methodological flaws as well as deficiencies in transparency and reporting in the current conduct of ML-based studies for treatment prediction. EHR data shares many of the same data quality issues as other types of data used in ML prediction, plus some unique challenges. To fully leverage EHR data’s power for patient stratification, increased attention to data quality and collection of patient-reported outcome data is needed.

## Introduction

Precision psychiatry proposes to tailor the diagnosis and treatment of psychiatric disorders to an individual’s unique profile of observable traits and biomarkers. In the decade since the National Institutes of Mental Health introduced the Research Domain operational Criteria framework [1], empirical evidence has demonstrated that disorders defined by DSM or ICD’s categorical criteria are heterogenous in underlying etiology, presenting symptoms, and response to treatment. By matching patients to the interventions with the highest likelihood of response based on their individual characteristics, the precision approach aims to transition clinical practice away from its one-size-fits-all strategy, whereby first-choice interventions are determined by mean treatment effects for categorically defined disorders.

Machine learning (ML) has generated considerable excitement for its potential to advance biomarker discovery and improve treatment outcomes. ML algorithms can adapt flexibly to large, high-dimensional, and noisy data. Compared to classical statistical methods, ML is more effective at handling non-linearities and interactions among many variables. To date, hundreds of proof-of-concept studies attest to the theoretical promise of ML for treatment prediction across a wide spectrum of biomarkers and interventions [2]. This considerable promise is overshadowed by a stark reality: no ML prediction tools have successful transitioned from research into widespread clinical practice. In fact, as reviewed here, validation of prediction models in independent data—the critical next step toward implementation—rarely occurs [3–5].

Data available for treatment prediction vary widely in quantity and quality. Clinical trial and other research data are meticulously curated, highly granular, but often modest in sample size. Data collected passively for other purposes, such as EHR and insurance claims, are copious but noisy. To date, most published prediction models repurpose clinical trial data (~70% in one estimate) with sample sizes inappropriate for data-hungry ML algorithms [3]. Few studies employ EHR data for treatment prediction, despite representing a wealth of real-world, continuously updated, longitudinal data on treatment trajectories for millions of patients [2].

## Stratified psychiatry

**Fig. 1:**
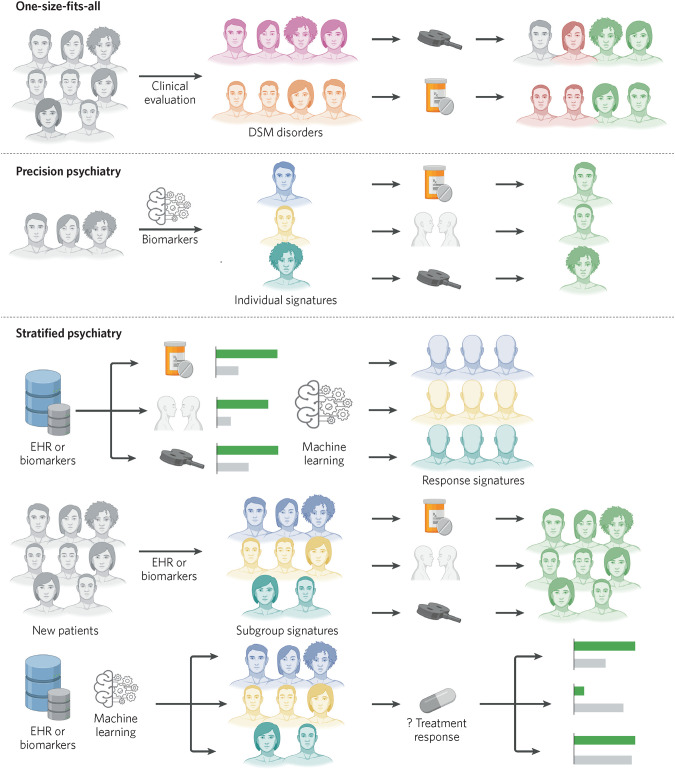
One-size-fits-all vs. precision vs. stratified psychiatry. In one size-fits-all psychiatry (top panel), patients are assigned to categorical disorders (e.g., DSM, ICD) by clinical evaluation and patient-reported symptoms. The evidence base (e.g., clinical trial data) largely determines first-choice treatment. A variable range of responses are observed (e.g., positive (green), no response (gray), or adverse response/effects (red)). In precision psychiatry (middle panel), the individual’s unique profile of biomarkers is matched to the exact treatment predicted for maximal response. In stratified psychiatry (bottom panel), machine learning may be employed using electronic health record (EHR) data or biomarker data to identify signatures associated with response to established interventions for a disorder. New patients may then be stratified to the treatment with high predicted response for their subgroup signature. Additionally, patients may be stratified by their shared EHR markers or biomarkers and prospectively treated to determine response, either to validate a response signature or establish association between signatures for a new or existing treatment. Figure created with BioRender.com.

One conception of precision psychiatry is individual-level treatment prediction using biomarkers to match each person to a specific intervention (from all available options) in a disorder-agnostic fashion (Fig. 1). Stratified psychiatry seeks to subgroup patients using their shared characteristics to increase likelihood of response to existing, approved treatments for a given disorder [6, 7]. The method leverages all markers that capture any significant inter-individual variation in response to different treatments—within and between modalities—including partial, non-response, and adverse reactions. Eliminating treatments likely to lead to no or adverse response for a subgroup increases the overall chance for response in selecting from the remaining options. Furthermore, by restricting predictions to established options with comparable efficacy for a disorder, harm from incorrect predictions may be lessened.

To illustrate, Arns et al. identified EEG biomarkers predicting response and non-response among patients randomized to receive escitalopram, sertraline, or venlafaxine for depression in the International Study to Predict Optimized Treatment (iSPOT-D) [8, 9]. No significant differences were observed for group-level clinical efficacy. Right frontal alpha asymmetry (FAA), however, predicted response and remission to escitalopram and sertraline but not venlafaxine for females only. Simulation suggested that stratifying the iSPOT-D patients to the three antidepressants using FAA could yield a 7–14% higher remission rate [9].

Reliable and reproducible biomarkers have proven elusive with data collection that is often slow, costly, and limited in size and scope. EHR data is rich with routinely collected qualitative and quantitative data for use in stratified psychiatry. Supervised and unsupervised learning can be used retrospectively to identify shared predictors (multi-dimensional signatures) of treatment response for a wide array of interventions at an unparalleled scale. These signatures can then be validated prospectively or by simulation. Patients with a shared signature are anticipated to possess common underlying biology and increased homogeneity of clinical outcomes. For example, Fabbri et al. found that treatment-resistant depression subgroups derived from EHR data strongly overlapped polygenic risk scores for major depressive disorder [10]. New patients may be classified and treated according to the similarity of their signature to established signatures [6]. Additionally, phenotypes derived from unsupervised learning of EHR data can be used prospectively to stratify patients for large-scale, pragmatic clinical trials of new or existing interventions to increase available response data.

## Interrogating the translational gap

Development of an ML prediction model involves a multi-step process [11]. Briefly, labeled data are partitioned into training and test subsets. The data subsets undergo preprocessing to minimize the impact of dataset anomalies (e.g., missing values, outliers, redundant features) on the algorithm’s learning process. The algorithm is applied to the training data, learning the relationship between the features and predictive target. Performance is typically evaluated via cross-validation to estimate the model’s performance on new observations (internal validation). However, this only approximates a model’s ability to generalize to unseen data. Prediction models must demonstrate the ability to generalize to independent datasets (external validation) [12]. Ideally, external validation should occur in a separate study by a different analytic team [13]. Clinical validation involves assessing a model’s generalization to real world data as well as potential clinical utility and impact. Randomized cluster trials, for instance, evaluate groups of patients randomly assigned to receive care based on a model’s prediction versus care-as-usual.

Few examples exist of predictive ML models advancing to clinical validation in psychiatry, indicative of a sizeable translational gap. Delgadillo et al. compared the efficacy and cost of stratified care compared to stepped care for a psychological intervention for depression (*n* = 951 patients) in a cluster randomized trial [14]. The investigators previously developed a ML prediction model to classify patients as standard or complex cases using self-reported measures and sociodemographic information extracted from clinical records (*n* = 1512 patients) [15]. In the prospective trial, complex cases were matched to high-intensity treatment and standard cases to low-intensity treatment. Stratified care was associated with a 7% increase in the probability of improvement in depressive symptoms at a modest ~$140 increase in cost per patient [14].

### Methodological flaws

What is driving this translational gap? Much of it may relate to challenges in generalizing models beyond their initial training data. There are no silver bullets in the development of ML prediction models and many potential pitfalls. The most common are overfitting and over-optimism due to insufficient training data, excess complexity improper (or lack of) cross-validation, and/or data leakage [16–18].

Most published ML studies in psychiatry suffer these methodological flaws [3–5]. Tornero-Costa et al. reviewed 153 ML applications in mental health and found only one study to be at low risk of bias by the Prediction model Risk Of Bias ASsessment Tool (PROBAST) criteria [3]. Approximately 37.3% of studies used a sample size of 150 or less to train models. Details on preprocessing were completely absent in 36.6% of studies and 47.7% lacked a description of data missingness. Only 13.7% of studies attempted external validation. Flaws in the analysis domain (e.g., attempts to control overfitting and optimism) contributed significantly to bias risk in most applications (90.8%). Furthermore, in 82.3% of the studies, data and developed model were not publicly accessible. Two other systematic reviews also found overall high risk of bias (>90%) among ML prediction studies, including poor reporting of preprocessing steps as well as low rates of internal and external validation [4, 5]. Meehan et al. additionally reported that only 22.7% of studies (of those meeting statistical standards) appropriately embedded feature selection within cross-validation to avoid data leakage [5].

The precise degree to which published ML prediction models overestimate their ability to generalize is difficult to estimate. In the area of prognosis prediction, Rosen et al. assessed 22 published prediction models of transition to psychosis in individuals at clinical high-risk [19]. Models were assessed for external validation from a multisite, naturalistic study. Only two models demonstrated “good” (AUC > = 0.7) performance and 9 models failed to achieve better than chance (AUC = 0.5) prediction. None of the models outperformed the clinician raters (AUC = 0.75) [19].

The model development process is vulnerable to human inductive biases, which can inflate model performance estimates due to unintentional errors or deliberate “gaming” for publication [17, 20]. Performance scores have become inappropriately prioritized in peer review due to erroneous higher = better assumptions. Most studies employ a single algorithm without justifying its selection or compare multiple algorithms’ performance on the same dataset, then select the best performing one (multiple testing issue) [17, 21]. Software packages like PyCaret (Python) offer the ability to “screen” the performance of a dozen or more algorithms on a dataset in a single step. This analytic flexibility creates risk, because even random data can be tuned to significance solely through manipulation of hyperparameters [17].

### Low quality or biased training data

Methodological shortcomings offer only partial explanation for the observed translational gap. As the saying goes, “garbage in, garbage out.” Low quality, small, or biased training data can generate unreliable models with poor generalization to new observations or worse, make unfair predictions that adversely impact patients. Ideal ML training data is large, representative of the population of interest, complete (low missingness), balanced, and possesses accurate and consistent feature and predictive target labels or values (low noise). Per the systematic reviews above, these data quality criteria have been often neglected [3–5].

EHR data share many of the same quality issues impacting data collected explicitly for research, as well as some unique challenges that have deterred its use for ML in the past [22–24]. EHR data are highly heterogenous, encompassing both structured and unstructured elements. Structured data is collected through predefined fields (e.g., demographics, diagnoses, lab results, medications, sensor readings). Unstructured data is effectively everything else, including imaging and text. Extracting meaningful features from unstructured EHR data is non-trivial and often requires supervised and unsupervised ML techniques.

The quality of EHR data can vary by physician and clinical site. Quality challenges with EHR data that can adversely impact ML models for stratified psychiatry include:

#### Selection bias

EHR populations are non-random samples, which may create differences between the training data population and the target population [25]. Patients with more severe symptoms or treatment resistance may be frequently referred. Factors other than need for treatment (e.g., insurance status, referral, specialty clinics) can lead to systematic overrepresentation or underrepresentation of certain groups or disorders in the data. Marginalized populations, such as racial and ethnic minorities, for example, face barriers to accessing care and may be absent in the data [26]. When an algorithm trains on data that is not diverse, the certainty of the model’s predictions is questionable for unrepresented groups (high epistemic uncertainty) [27]. This may lead to unfair predictions (algorithmic bias) [28].

#### Missingness

Missing data are common in EHRs. The impacts of missing data on model performance can be severe, especially when the data are missing not at random or missing at random but with a high proportion of missing values [29]. Furthermore, the frequency of records can vary substantially by patient. One individual may have multiple records in a period, others may have none [30]. Does absence of a diagnosis indicate true lack of a disorder or simply reflect that the patient received care elsewhere during a given interval? Structured self-reported patient outcome measures (e.g., psychometric measures) are often missing or incomplete [31].

#### Inaccurate features and targets

Feature and target labels or values provide the ground truth for learning. Inaccuracies and missingness generate noise, which can hinder effective learning. The lineage of a given data element is important in considering its reliability and validity. For example, a patient’s diagnoses may be extracted from clinical notes, encounter/billing data, or problem lists (often not dated or updated) [32]. In some cases, the evaluating practitioner enters the encounter-associated diagnostic codes; in other instances, these are abstracted by a medical billing agent, creating uncertainty.

#### Inconsistency

Imaging and sensor-based data may be collected using different acquisition parameters and equipment, leading to variability in measurements across EHRs and over time [33]. Data may be collected using different coding systems (e.g., DSM, ICD), the criteria for which also change over time. These issues can hinder external validation as well as contribute to data drift with the potential for deterioration in model performance [34].

#### Imbalanced data

When data are imbalanced, ML classification models may be more likely to predict the majority class, resulting in a high accuracy but low sensitivity or specificity for the minority class [35]. The consequences of data imbalance can be severe, particularly when the minority class is the most clinically relevant (e.g., patients with suicidal ideation who go on to attempt, adverse drug reactions).

#### Temporal dynamics

Patient records represent a sequence of events over time [36]. Diagnostic clarification may create conflicts (e.g., depression later revealed to be bipolar disorder), depending on the forward and lookback windows used to create a dataset. Failure to appropriately account for the longitudinal nature of a patient’s clinical course can contribute to data leakage. Temporal data leakage occurs when future information is inadvertently used to make predictions for past events (e.g., including a future co-morbidity when predicting response to past treatment). Feature leakage occurs when variables expose information about the prediction target.

Empirical evidence indicates that preprocessing techniques can just as easily mitigate as exacerbate underlying data quality and bias issues. For example, missing data may be handled by complete case analysis (i.e., removal of observations with missing features) or imputation [37]. If data are not missing completely at random, deletion may eliminate key individuals [29]. Fernando et al. found that records containing missing data tended to be “fairer” than complete records and that their removal could contribute to algorithmic bias [38]. In the case of imputation, if the estimated values do not accurately represent the true underlying data, replacing “missing” values may inject error (e.g., imputing scores for psychometric scale items absent due to skip logic) and impact feature selection [39].

EHR data often require the creation of proxy features and outcomes to capture concepts (e.g., continuous prescription refills as an indicator of treatment effectiveness) or to reduce feature and label noise [40, 41]. No standards currently exist to guide such decisions or their reporting, creating high risk for bias. For example, if attempting to determine cannabis use when a patient was treated with a given antidepressant, one could check for a DSM/ICD diagnosis in their encounters or problem list, mine clinical notes to see whether use was endorsed/denied, or examine urine toxicology for positive/negative results. Each choice carries a different degree of uncertainty. Absence of evidence does not indicate evidence of absence [42], although studies often make that assumption.

## Call for improvements

Coupling EHR data with a stratified approach is a promising step toward precision psychiatry with the potential to improve treatment outcomes without development of new treatments or collecting new data. This path, however, requires a commitment to improving EHR data quality and addressing known challenges. In ML, data is often treated as a “fixed” entity with quantity assumed paramount over quality [43]. In model-centric ML, noise is iteratively “tuned” out through hyperparameter adjustment and increasing the complexity of the model architecture. A data-centric approach contends that high-quality training data best improves model performance and generalization. The model is “fixed,” and the data is iteratively optimized through thoughtful preprocessing [44].

Advancement requires that researchers, funders, and journals prioritize the assessment and reporting of data quality and preprocessing methodology for EHR data and derived markers as highly as they do for biological and imaging biomarkers [45]. Automated assessment tools and reporting guidelines and instruments are needed [46], especially given the push toward federated learning that would see models but not data exchanged for external validation to address pressing privacy concerns [47]. Exciting developments in the use of autoencoders and natural language processing techniques for the automated extraction of features from all types of EHR data at scale can help increase standardization, but these also require validation [48, 49]. Finally, no strategy can optimize data that is simply missing en masse. Proxy treatment response measures are a poor substitute for patient-reported outcomes. There is an urgent need for increased implementation of patient-reported outcome measures in EHRs, which is often impeded by concerns regarding workflow disruption, thresholds for action, logistical/technical barriers, and lack of incentives for practitioners or patients [50].

## Conclusions

The hype surrounding ML is substantial, but its potential to harness the power of big data in the service of precision psychiatry cannot be ignored. Stratified psychiatry, underpinned by the wealth of existing information within EHRs, can propel the field forward. However, we can no longer ignore methodological and data quality issues and expect to close the translational gap. Laxity in methodological rigor, reporting standards, and external validation must be addressed. As precision psychiatry continues to evolve, the integration of ML and EHRs will be instrumental in translating the promise of personalized care into a tangible clinical reality.
